# Supplementary material for: Distribution of Small Ruminant Lentivirus Genotypes A and B in Goat and Sheep Production Units in Mexico
Source: Vet Sci. 2025 Mar 1;12(3):204. doi: 10.3390/vetsci12030204 (PMC11946063; doi:10.3390/vetsci12030204)
Supplement: Supplementary file 1 [file vetsci-12-00204-s001.zip › vetsci-3392920-supplementary.pdf]

Supplementary Materials

Table S1. The risk factors identified for each analyzed group, together with the chi-square (χ^2) values and odds ratios (ORs), demonstrate the association and probability of seropositivity and molecular detection or genotyping of the presence of SRLV in groups exposed to the risk factor compared with those not exposed in the flocks.

Risk factor	Cluster	χ^2	*n*	*p*	OR	CI (95%)	
						Lower	Upper
**Positive via ELISA**							
Age	Goats	40.545	384	1.921e-10	5.567043	3.254123	10.00241
	Central region	62.884	412	2.193e-15	5.864016	3.756532	9.312927
	Milk production	20.264	266	6.745e-06	3.951368	2.189377	7.456644
	Intensive system	41.521	568	1.166e-10	4.25298	2.719005	6.799602
	Semi-intensive system	35.179	322	3.007e-09	8.137532	3.899447	18.39781
Flock size	Goats	4.5059	384	0.03378	1.918159	1.081966	3.422343
	Central region	17.987	412	2.224e-05	2.614197	1.689509	4.102535
	Northern region	21.01	278	4.569e-06	4.313877	2.323073	8.50667
	Intensive system	49.278	568	2.221e-12	4.17961	2.793625	6.374095
Veterinary assistance	Southern region	7.3013	200	0.006891	4.294696	1.602319	15.29382
	Meat	6.6062	624	0.01016	2.001198	1.212989	3.450629
	Semi-intensive system	5.9765	322	0.0145	2.320354	1.232053	4.664864
**Positive via LTR-PCR**							
Age	Goats	6.7189	384	0.00954	3.502318	1.454434	10.56918
	Central region	9.127	412	0.002519	2.868096	1.49596	5.99505
	Intensive system	5.1973	568	0.02262	2.201461	1.176272	4.522961
Flock size	Goats	6.2003	384	0.01277	2.716389	1.248687	5.617172
	Central region	9.884	412	0.001667	2.423232	1.407964	4.172297
	Intensive system	34.792	568	3.668e-09	3.709826	2.380355	5.816902
Mixed flock	Central region	12.221	412	0.0004726	3.316306	1.732065	6.925714
Producer knows about SRLV diseases	Sheep	6.3274	507	0.01189	1.944454	1.173091	3.192149
Contact with other flocks	Sheep	7.3143	507	0.006841	2.206286	1.257549	3.835165
**Genotype A-Positive via Real-Time PCR**							
Flock size	Semi-intensive system	8.809	323	0.002997	7.067084	2.057798	47.95697
**Genotype B-Positive via Real-Time PCR**							
Age	Goats	5.4883	384	0.01914	3.178518	1.311478	9.624881
	Central region	63.364	412	1.718e-15	18.54966	8.096508	54.38504
	Intensive system	37.162	568	1.087e-09	10.64438	4.68797	31.01246
Flock size	Goats	7.7406	384	0.005399	3.045691	1.388801	6.371866
	Sheep	17.114	507	3.52e-05	2.146647	1.503195	3.077296

Risk factor	Cluster	χ^2	*n*	*p*	OR	CI (95%)	
						Lower	Upper
	Central region	63.233	412	1.836e-15	6.190697	3.897266	9.957912
	Northern Region	27.744	279	1.385e-07	4.358709	2.512236	7.674759
	Southern region	20.691	200	5.398e-06	4.470929	2.352427	8.730779
	Meat	12.208	625	0.0004759	1.792921	1.299993	2.477964
	Intensive system	203.19	568	< 2.2e-16	19.34179	12.35473	30.90448
Mixed flock	Sheep	67.568	507	< 2.2e-16	4.915188	3.348758	7.300681
	Meat	62.586	625	2.551e-15	3.892513	2.774841	5.503109
	Intensive system	27.092	568	1.94e-07	3.394871	2.15031	5.54769
	Semi-intensive system	20.677	323	5.437e-06	3.066697	1.89975	4.987989
Producer with technical training	Southern region	20.691	200	5.398e-06	4.470929	2.352427	8.730779
	Semi-intensive system	4.126	323	0.04223	1.741165	1.045063	2.894016
Producer knows about SRLV diseases		20.691	200	5.398e-06	4.470929	2.352427	8.730779
	Southern region	6.2857	323	0.01217	2.045073	1.191355	3.510294
	Semi-intensive system	28.686	507	8.513e-08	2.686174	1.876726	3.86486
Participation in livestock fairs	Sheep	14.318	412	0.0001544	2.371153	1.529605	3.715016
	Central region	24.869	625	6.137e-07	2.30056	1.6632	3.192443
	Meat	30.459	323	3.41e-08	4.023878	2.449408	6.680498
	Semi-intensive system	10.605	507	0.001128	2.593788	1.482913	4.673156
Contact with other flocks	Sheep	20.691	200	5.398e-06	4.470929	2.352427	8.730779
	Southern region	6.2857	323	0.01217	2.045073	1.191355	3.510294
	Semi-intensive system	6.2857	323	0.01217	2.045073	1.191355	3.510294
Unhealthy animal isolation	Sheep	5.9978	507	0.01432	1.843334	1.151125	2.980273
	Southern region	20.691	200	5.398e-06	4.470929	2.352427	8.730779
	Semi-intensive system	4.7405	323	0.02946	1.75093	1.080062	2.837938
Veterinary assistance	Goats	4.6719	384	0.03066	2.309507	1.138777	5.013168
**Genotype A/B-Positive via Real-Time PCR**							
Flock size	Northern region	4.3447	279	0.03712	2.258545	1.09957	4.772971
Producer with technical training	Sheep	13.45	507	0.000245	6.920352	2.505484	29.6719
	Northern region	23.187	279	1.47e-06	11.74387	4.042805	51.7384
	Meat	20.174	625	7.07e-06	9.284586	3.404423	39.54187
Unhealthy animal isolation	Sheep	7.0915	507	0.007745	3.467443	1.488183	10.26167
	Northern region	15.463	279	8.413e-05	4.935802	2.233421	12.16258
	Meat	9.7335	625	0.001809	3.224648	1.607556	7.447448
Veterinary assistance	Intensive system	30.383	568	3.547e-08	8.138561	3.722114	21.6316

CI= confidence interval; *p* = *p* value; *n* = observations.
